# Study on the Influencing Factors of Farmers’ Adoption of Conservation Tillage Technology in Black Soil Region in China: A Logistic-ISM Model Approach

**DOI:** 10.3390/ijerph19137762

**Published:** 2022-06-24

**Authors:** Hongpeng Guo, Wenkai Zhao, Chulin Pan, Guijie Qiu, Shuang Xu, Shun Liu

**Affiliations:** College of Biological and Agricultural Engineering, Jilin University, 5988 Renmin Street, Changchun 130022, China; ghp@jlu.edu.cn (H.G.); zhaowk20@mails.jlu.edu.cn (W.Z.); pancl@jlu.edu.cn (C.P.); xushuang_jlu@163.com (S.X.); liushun@jlu.edu.cn (S.L.)

**Keywords:** black soil region, conservation tillage, farmers’ behavior, Logistic-ISM model

## Abstract

The adoption of conservation tillage technology can improve the production efficiency of black soils (mollisols), and it has great significance to ensure the sustainable development of agriculture. This paper takes farmers in the black soil region of Jilin Province as the research object, uses 442 survey data of farmers in seven municipal areas in the black soil region of Jilin Province, constructs a logistic-ISM model, first determines the influencing factors of farmers’ adoption of conservation tillage technology, and then analyzes the hierarchical structure of each influencing factor. The results show that: (1) among the eight significant influencing factors of farmers’ adoption of conservation tillage technology, age, whether they know the government’s subsidies for conservation tillage and the number of labor force are the deep-rooted factors; (2) Education level, whether you know that the government is promoting conservation tillage, and the planting area are intermediate level factors; (3) whether they have received the technical services of conservation tillage and whether the cultivated land is scattered is the direct factors. Based on the significance analysis of the influencing factors of farmers’ adoption of conservation tillage technology and the research on the action mechanism of the influencing factors of farmers’ adoption of conservation tillage technology, this paper puts forward policy suggestions to improve the extension system of conservation tillage technology, improve the implementation of land transfer and subsidy policies, strengthen the ability of rural socialized services, and strengthen the publicity of black soils protection.

## 1. Introduction

Land is the material basis for human survival and development, and is a valuable non-renewable resource. The black soils distributed in the middle and high latitudes of the world are recognized as the most fertile soil in the world. It has the characteristics of good properties, high fertility and suitable for farming. It is a soil resource worthy of protection and plays an important role in global agricultural production [1]. The Mississippi plain of the United States, the Ukrainian plain of Ukraine and the Northeast Plain of China are the three major black soil regions in the world [2]. The black soil region of Northeast China Plain covers a total area of about 1.09 million square kilometers, mainly distributed in Liaoning Province, Jilin Province, Heilongjiang Province and the east of Inner Mongolia Autonomous Region [3]. The black soil region of Jilin Province is a critical grain producing area in China. Its productivity plays an indispensable role in ensuring Chinese food security [4]. Due to the long-term intensive cultivation and the lack of effective protection methods, the black soils in Jilin Province have undergone soil degradation, mainly manifested in the shrinkage of the black soil region, the sharp reduction in organic matter in the black soils layer, the thinning of the tillage layer and the thickening of the plough bottom, and the degradation of biological functions [5,6,7,8]. According to the statistical data released by China’s Ministry of Agriculture and Rural Affairs in 2017, the content of soil organic matter in the cultivated layer of black soils in Northeast China has decreased by 1/3 in the last 60 years, with a decrease of 50% in some areas, and the content of soil organic matter has been less than 20 g/kg. Northeast China is a very serious area of soil and water loss. The erosion area is expanding, the intensity is increasing, and the number of erosion ditches is increasing, resulting in the continuous decline of soil nutrients. Soil erosion has become an urgent problem in the black soil region of Northeast China [9]. This has seriously affected the sustainable development of agriculture in the black soil region of Northeast China, reduced the production efficiency of cultivated land, weakened the improvement of grain production capacity, and threatened the national food security. Practice has proved that conservation tillage is an effective way to protect and utilize black soils resources in Northeast China [10].

Conservation tillage is an environment-friendly soil tillage model [11]. There is no unified concept in the world. It first rose in the United States in the 1930s. It is a modern dry farming technology of sustainable agriculture and is widely used all over the world [12,13,14,15,16]. The definition of conservation tillage given by the Conservation Tillage Information Center is based on the coverage rate of crop straw residues. It is considered that after the completion of one season of crops, the surface stubble coverage of more than 30% is conservation tillage. The main crops straw treatment methods in the black soil region of Northeast China are straw deep ploughing, straw crushing and less tillage or no tillage + stubble mulching [17]. The results of field measurements and simulation tests show that, compared with traditional tillage, conservation tillage can reduce wind erosion by 30–70% and surface runoff by 40–80%. The amount of stubble cover is an important factor to reduce soil erosion by wind and water. The soil is still exposed more in the way of straw deep ploughing, and the soil is exposed least in the way of less tillage or no tillage + stubble cover, so the effect of soil and water conservation is also the best [18,19]. The FAO has put forward three principles for conservation tillage: minimum mechanical soil disturbance, permanent soil organic cover and species diversity. These three interrelated principles can improve agricultural productivity and ensure the sustainable development of agriculture [20]. In order to achieve the above objectives, scholars have summarized several specific measures. Conservation tillage is defined as a sustainable agricultural technology through comprehensive supporting measures such as less tillage, no tillage, surface micro-topography modification technology, surface coverage and rational planting, in order to reduce farmland soil erosion, protect farmland ecological environment, and obtain the coordinated development of ecological, economic and social benefits [21,22]. Its core technologies can be summarized into four aspects: less tillage or no tillage sowing, straw returning to the field, subsoiling and integrated pest control, it can not only improve soil properties, improve soil organic matter content and water storage capacity, reduce soil wind and water erosion [23,24,25], but also reduce greenhouse gas emissions, reduce energy consumption and inhibit cultivated land degradation [26,27]. It also increases crop yield [28,29], which can realize the effective utilization of cultivated land resources.

Over the years, the Chinese government’s cultivated land protection policy has achieved some results, but it is not deep enough at the level of farmers. Too macro policies will easily become a mere formality because farmers are the main users and protectors of land, and whether the black soils can be effectively protected depends on the behavior of farmers [30,31]. Scholars have conducted extensive research on the factors affecting the adoption of conservation tillage technology by farmers. There are many factors affecting farmers’ adoption of conservation tillage technology, mainly including personal and family characteristics, cultivated land characteristics, farming cognition and external factors [32]. The conditions of farmers themselves and their families are an important aspect affecting the adoption of conservation tillage technology. Guo et al., studied the behavior and willingness of farmers to apply biopesticides. The results show that education, rice planting scale, cognition of biopesticides, social norms and other factors will affect farmers’ adoption of biopesticides [33]. Sheikh et al., believe that when adopting no tillage technology, personal characteristics are the main influencing factors, including education level, identity of renters, attitude towards new technology risks and technology promotion of extension personnel [34]. Featherstone et al., studied farmers in Kansas and concluded that older farmers had less long-term investment in conservation tillage [35]. D’Emden et al., believe that the higher education level of growers and the more comprehensive their understanding of no tillage technology, the more likely they are to adopt no tillage behavior [36]. Greiner et al., conducted an empirical study on farmers in northern Australia. Their protection behavior is less affected by financial, economic and social factors, but greatly affected by moral standards [37].

Natural condition factors mainly refer to the natural conditions of cultivated land. Cultivated land area, fragmentation degree and slope of cultivated land, whether there is transferred land and so on are the main factors affecting the adoption of farmers’ conservation tillage technology. Bekele et al., analyzed the influencing factors of farmers’ water and soil protection behavior in eastern Ethiopia, and concluded that the cultivated land area and slope are positively correlated with farmers’ protection behavior [38]. Si et al., used data from the Gansu Province, and showed that the signing of land transfer contracts helps farmers decide to adopt conservation tillage technology [39]. Xu et al., studied the influencing factors of land fragmentation in Jiangsu Province, China. Land fragmentation has an important impact on agricultural production. It will lead to low agricultural production efficiency, increased production costs and difficulties in agricultural mechanization, which is not conducive to the sustainable development of cultivated land [40].

External factors mainly include social network, social capital, government policies, government subsidies, technical training and so on. Li et al., and others used the data of 1237 farmers in the Loess Plateau to analyze the regulatory role of government subsidies in soil and water conservation of cultivated land. The results showed that government subsidies can make up for the possible risk losses of farmers, so they can encourage farmers to adopt soil and water conservation farming technology [41]. The research of Kurkalova et al., shows that due to the uncertainty of income and risk, the subsidy of conservation tillage has an important impact on farmers’ decision making whether to adopt conservation tillage [42]. Singh et al., studied small-scale farmers who manage land under uncertainty and risk, and how the dynamics of farmers’ family interactions, social network, environment, system and market will affect their agricultural decision making [43]. Burton et al., studied the behavior of European farmers in voluntary agricultural environmental programs, and the results showed that profitable agricultural environmental protection projects will more effectively change long-term behavior [44]. Using the method of literature analysis, Stupak et al., investigated in detail the changes of agricultural soil protection system in Ukraine and discussed its impact on the behavior of agricultural producers and specific soil protection measures. The results show that the instability of policies and institutional changes will affect the behavior of agricultural practitioners [45].

To sum up, although great progress has been made in the research on the influencing factors of farmers’ new technology adoption and decision making, there are also some deficiencies: on the one hand, in Jilin Province, as an important part of China’s black soil region and an important grain producing area, no scholars have conducted in-depth research on the influencing factors of farmers’ conservation tillage technology adoption; On the other hand, researchers focus on the judgment of significant influencing factors and the analysis of influence degree, or the analysis of farmers’ decision making willingness and behavior of new technology, and lack of in-depth mining of the relationship and hierarchy between various factors.

Based on the questionnaire data of farmers in several counties and cities of Jilin Province, this paper uses a logistic model to analyze the influencing factors of farmers’ adoption of conservation tillage technology, and then further analyzes the correlation and hierarchical structure between the influencing factors through an ISM model to explore the internal mechanism affecting the adoption of conservation tillage technology. This is with the aim to better promote conservation tillage technology, improve cultivated land production efficiency and sustainable development of cultivated land, and provide theoretical support and specific suggestions for the release and implementation of relevant government policies.

## 2. Materials and Methods

### 2.1. Data

#### 2.1.1. Description of Study Area

Jilin Province is located in the middle of Northeast China. Affected by the temperate monsoon climate, it is hot and rainy in the summer and cold and rainy in the winter. Herbs grow rapidly in the summer, but decay slowly in the winter. Plant litter accumulates in the soil, forming a fertile humus layer in the black soil region over time. Black soils are also known as isohumisols in Chinese soil taxonomy or mollisols in USA soil taxonomy [46].

Jilin Province is located in the black soil region of Northeast China, with a black soil cultivated land area of 4.49 million hm^2^, accounting for 64% of the cultivated land area of the province. Jilin Province is an important grain producing area and has always been China’s main commodity grain export province. The annual grain commodity rate exceeds 80%, the commodity grain provided accounts for 10% of the country and the reserve grain accounts for 20% of the country. For more than 20 consecutive years, the amount of grain transferred out ranks first in the country and has made great contributions to China’s food security.

Jilin Province has always attached importance to the protection of black land. Conservation tillage has long been vigorously promoted through policy subsidies, technology subsidies and other forms. The main technologies to be promoted include less or no tillage, straw residue coverage, straw crushing, deep ploughing, subsoiling, ridge farming, increased application of organic fertilizer, and soybean rotation. According to the information released by the Ministry of Agriculture and Rural Affairs of China, by the end of 2020, the area of conservation tillage in Jilin Province has increased from 0.3 million hm^2^ in 2015 to 1.24 million hm^2^. There are more and more farmers adopting conservation tillage technology in Jilin Province. They feel good after using conservation tillage technology.

#### 2.1.2. Data Collection and Sample

The data used in this paper come from a questionnaire survey conducted by the research group in the black soil region of Jilin Province, China from November 2020 to January 2021. Farmers’ samples are selected according to typical sampling methods: firstly, according to the black soil region of conservation tillage, seven counties and cities in Jilin Province are selected, including Changchun City, Siping City, Songyuan City, Jilin City, Liaoyuan City, Tonghua City and Baicheng City. Then, two townships were randomly selected in each county and city. Finally, four natural villages were selected in each township and 8–10 farmers in each village for investigation. A total of 496 questionnaires were distributed. After all the questionnaires were recovered and carefully checked and processed, 54 invalid questionnaires were eliminated, and 442 valid farmers’ data were finally formed. The effective rate of the questionnaire was 89.1%.

#### 2.1.3. Variables and Measure

It can be learned from the preliminary research that the farmers in the black soil region of Jilin Province are mainly operated by families. Most of the investigated farmers are new types of agricultural operating entities, with an average planting area of about 13 hm^2^ per household and a median of 4 hm^2^, including their own land and land transferred from others. In terms of formal meaning, these farmers are small-scale and belong to small farmers. In China, the view that the behavior of small farmers advocated by Popkin and Schultz is economic rationality is relatively in the mainstream [47]. Farmers are very rational individualists. They are rational economic people who make reasonable production choices in pursuit of the best interests [48]. According to this theory, combined with the relevant research of other scholars mentioned above, this paper analyzes the influencing factors of farmers’ adoption of conservation tillage technology from four aspects: Farmers’ individual and family characteristics, cultivated land characteristics, cognition of conservation tillage and external conditions. The selection of specific variables and their expected influence directions are as follows.

The personal and family characteristics of farmers mainly include the age, gender and education level of the surveyed farmers, whether they have a part-time job, labor force population and the years of planting. With the growth of farmers’ age, their ability to accept new things will be reduced, and they will instinctively resist new technologies. Men are better suited than women to field work, which is a physically demanding job, so men are more likely to use conservation tillage techniques. Farmers with a high level of education will know and pay more attention to conservation tillage, and they will master new technologies faster, so they are more likely to adopt conservation tillage technology. The part-time income of farmers who go out to work will be higher than that of agriculture. Conservation tillage can reduce the input of labor force, give farmers more leisure time to earn money from employment other than farming, so they are more likely to choose conservation tillage. The amount of the labor force population reflects the labor volume of farmers’ families, which may have an impact on the adoption of conservation tillage technology. The longer the planting years, the more fixed the thinking, the lower the acceptance of new things, so it is more impossible to adopt conservation tillage technology. Therefore, this paper assumes that the increase in age and years of planting have a negative impact on the implementation of conservation tillage; the male gender, part-time employment and high education level of farmers have a positive impact on conservation tillage; the impact direction of labor force population is unknown.

The characteristics of cultivated land mainly include the planting area, the degree of fragmentation of land, whether the cultivated land has slope land, the types of crops planted, and the adopted fertility measures. The larger the planting area, the more fertile the land is, which is more conducive to the use of agricultural machinery. The more concentrated the cultivated land is, the more conducive it is to large-scale production. Different crops have different requirements for cultivated land, and the input of farmers may be different, so it may affect the adoption of farmers’ conservation tillage technology. This paper assumes that the planting area is large, the land is concentrated, and the cultivated land has no slope, which has a positive effect on farmers’ implementation of conservation tillage, and the influence direction of the types of crops are unknown.

Farming cognition is also one of the important factors in the implementation of conservation tillage. Whether they have received relevant knowledge training, that is, whether the villages and towns where the farmers are located have services of conservation tillage technology is also important. After understanding conservation tillage technology, they are more likely to adopt conservation tillage. Farmers applying organic fertilizer or returning straw to the field are obviously more inclined to adopt new technologies and are more likely to adopt Conservation Tillage Technologies. In addition, whether the following conservation tillage measures are known, such as less tillage and no tillage, straw residue mulching, straw crushing and mulching, deep ploughing or subsoiling, ridge farming, soybean rotation and other measures may be very imported. Farmers may implement some of the above measures based on agricultural experience or training. This paper assumes that after receiving the technical services of conservation tillage, adopting appropriate fertilization measures, and understanding the conservation tillage measures will have a positive impact on the implementation of conservation tillage.

External factors mainly refer to the policies and publicity of conservation tillage issued by governments at all levels. Farmers tend to trust the publicity of the government, so it is easier for farmers advocated by the government to adopt conservation tillage technology. Farmers are rational, economic people and will make choices to maximize their own interests. Relevant subsidies will encourage them to implement conservation tillage. Similarly, satisfaction with agricultural machinery services will also increase the possibility of adopting conservation tillage technology. Therefore, this paper assumes that there are conservation tillage subsidies, farmers are satisfied with agricultural machinery services, and have a positive effect on farmers’ implementation of conservation tillage.

The dependent variable of this study is whether farmers adopt conservation tillage technology, and the value of farmers adopting conservation tillage is 1; the value assigned to farmers without conservation tillage technology is 0. The independent variables mainly select four types of factors (personal and family characteristics, cultivated land characteristics, farming cognition and external factors) affecting farmers’ adoption of conservation tillage technology, a total of 16 variables. The details are shown in Table 1.

### 2.2. Methods

In this study, the results of whether farmers in the black soil region adopt conservation tillage technology are only adopted and not adopted, which is an obvious binary decision-making problem, that is, the variable value of conservation tillage technology is 1, and the variable value of non-conservation tillage technology is 0. Therefore, this paper uses a logistic regression model to explore which factors will affect farmers’ adoption of conservation tillage technology, and previous studies have also proved the applicability of a logistic regression model in the study of influencing factors [49,50,51]. The basic form of the model is as follows:

(1) $$\ln\left(\frac{p_{i}}{1-p_{i}}\right)=Y=\beta_{0}+\beta_{1}x_{1}+\beta_{2}x_{2}+\cdots +\beta_{i}x_{i}$$

In Formula (1), $p_{i}$ represents the probability of farmers implementing conservation tillage; Y is the dependent variable, indicating whether farmers implement conservation tillage; $\beta_{i}$ is the regression coefficient of influencing factors; $x_{i}$ is an independent variable, indicating the ith influencing factor; $\beta_{0}$ is the constant of the regression equation.

The factors affecting farmers’ adoption of conservation tillage technology may have internal relations. It is important to distinguish the relationship levels between factors and find out the core factors. This paper will use an ISM model for further analysis. The significant influencing factors obtained from the results of the logistic model are the constituent elements of the ISM analysis system, so as to realize the linkage application of logistic ism [52,53].

The interpretive structural model is a method developed by Professor Warfield in 1976 to analyze complex socioeconomic problems. Its basic core idea is to extract the constituent elements of the problem, use the incidence matrix principle in graph theory and computer technology to process the information of the factors and their mutual influence relationship, and finally construct the system into a multi-level hierarchical model with a good structural relationship [54,55]. Through the ISM model, we can obtain the multi-level hierarchical structure among the elements of the complex system, make many staggered elements organized and clear, and find the internal relationship between the main elements, so as to provide a reference for revealing the internal law of system structure and extracting useful information [56,57].

Assuming that there are k factors affecting farmers’ adoption of conservation tillage technology, using $S_{0}$ indicates whether farmers adopt conservation tillage technology, $S_{i}\left(i=1,2,3\ldots \ldots k\right)$ represents the influencing factors of farmers’ adoption of conservation tillage technology. The elements in the adjacency matrix R can be determined by Equation (2).

(2) $$R=\{\begin{array}{ll} 1 & S_{i}\;\mathrm{is}\;\mathrm{related}\;\mathrm{to}\;S_{j}\\ 0 & S_{i}\;\mathrm{is}\;\mathrm{not}\;\mathrm{related}\;\mathrm{to}\;S_{j} \end{array}$$

The adjacency matrix of these factors can be obtained. The adjacency matrix reflects the direct relationship between elements, the reachability matrix reflects the indirect relationship between elements, and the reachability matrix between factors can be obtained from Formula (3):

(3) $$M={\left(R+I\right)}^{\lambda +1}={\left(R+I\right)}^{\lambda }\neq {\left(R+I\right)}^{\lambda -1}\neq \cdots \neq {\left(R+I\right)}^{2}\neq \left(R+I\right)$$

In Formula (3), I is the identity matrix 2≤λ≤k. The power operation in the matrix adopts the Boolean algorithm.

The factors contained in the top layer can be determined according to Formulas (4) and (5):

(4) $$P\left(S_{i}\right)=\left\{S_{j}|m_{ij}=1\right\},Q\left(S_{i}\right)=\left\{S_{j}|m_{ji}=1\right\}$$

(5) $$L_{1}=\left\{S_{i}|P\left(S_{i}\right)\cap Q\left(S_{i}\right)=P\left(S_{i}\right);i=0,1,2\cdots ,k\right\}\;$$

In Formula (4) $m_{ij}$ and $m_{ji}$ refers to the factor of reachability matrix M. $P\left(S_{i}\right)$ is the reachable set, representing the set of all reachable factors starting from $S_{i}$ in the reachable matrix; $Q\left(S_{i}\right)$ is the antecedent set and represents the set of all factors that can reach factor $S_{i}$ in the reachable matrix.

## 3. Results

### 3.1. Logistic Regression

The data of this study were processed with stata15 software. As shown in Table 2, from the regression results, the two-fold logarithmic value is 415.837. The pseudo $R^{2}$ is 0.324, and the p is less than 0.0001, which is significant at the confidence level of 0.01, indicating that the overall fit of the model is good.

Through regression, eight variables are significant influencing factors, including age, education level, whether the government publicity about conservation tillage is known, whether they have received the technical service of conservation tillage, whether the government’s subsidies for conservation tillage are known; while the labor force, planting area and whether cultivated land is scattered have a significant negative impact. These eight significant influencing factors will be further analyzed.

### 3.2. ISM Regression

According to the steps of the ISM analysis method, the system composition is determined first. In this paper, $S_{1},S_{2},S_{3},S_{4},S_{5},S_{6},S_{7},S_{8}$ represent the eight significant variables in the results of the logistic model, namely, age, education level, whether the government publicity about conservation tillage is known, whether they have received the technical service of conservation tillage, whether the government’s subsidies for conservation tillage are known, labor force, planting area and whether cultivated land is scattered. Based on the analysis, discussion and consultation with relevant experts, scholars and managers of functional departments, the logical relationship between the eight influencing factors is obtained, and the logical relationship between the factors affecting farmers’ adoption of conservation tillage technology is given as shown in Table 3. Where “V” indicates that the row factor has an impact on the column factor, “A” indicates that the column factor has an impact on the row factor and “0” indicates that there is no interaction between the row factor and the column factor. By default, it has no impact on itself.

According to Table 3 and Formula (3), the adjacency matrix R between influencing factors is obtained in Formula (6).

(6) $$R=\begin{array}{c} S_{0}\\ S_{1}\\ S_{2}\\ S_{3}\\ S_{4}\\ S_{5}\\ S_{6}\\ S_{7}\\ S_{8} \end{array}\left[\begin{array}{ccccccccc} 0 & 0 & 0 & 0 & 0 & 0 & 0 & 0 & 0\\ 1 & 0 & 1 & 1 & 1 & 0 & 0 & 0 & 0\\ 1 & 0 & 0 & 0 & 1 & 0 & 0 & 0 & 0\\ 1 & 0 & 0 & 0 & 1 & 0 & 0 & 0 & 0\\ 1 & 0 & 0 & 0 & 0 & 0 & 0 & 0 & 0\\ 1 & 0 & 0 & 1 & 1 & 0 & 0 & 1 & 0\\ 1 & 0 & 0 & 0 & 0 & 0 & 0 & 1 & 0\\ 1 & 0 & 0 & 0 & 0 & 0 & 0 & 0 & 1\\ 1 & 0 & 0 & 0 & 0 & 0 & 0 & 0 & 0 \end{array}\right]$$

The reachability matrix M is calculated by Matlab R2021a software, as shown in Formula (7).

(7) $$M=\begin{array}{c} S_{0}\\ S_{1}\\ S_{2}\\ S_{3}\\ S_{4}\\ S_{5}\\ S_{6}\\ S_{7}\\ S_{8} \end{array}\left[\begin{array}{ccccccccc} 1 & 0 & 0 & 0 & 0 & 0 & 0 & 0 & 0\\ 1 & 1 & 1 & 1 & 1 & 0 & 0 & 0 & 0\\ 1 & 0 & 1 & 0 & 1 & 0 & 0 & 0 & 0\\ 1 & 0 & 0 & 1 & 1 & 0 & 0 & 0 & 0\\ 1 & 0 & 0 & 0 & 1 & 0 & 0 & 0 & 0\\ 1 & 0 & 0 & 1 & 1 & 1 & 0 & 1 & 1\\ 1 & 0 & 0 & 0 & 0 & 0 & 1 & 1 & 1\\ 1 & 0 & 0 & 0 & 0 & 0 & 0 & 1 & 1\\ 1 & 0 & 0 & 0 & 0 & 0 & 0 & 0 & 1 \end{array}\right]$$

According to the principle of Formula (5), the following can be obtained by using Matlab R2021a software: $L_{1}=\left\{S_{0}\right\},L_{2}=\left\{S_{4},S_{8}\right\},L_{3}=\left\{S_{2},S_{3},S_{7}\right\},L_{4}=\left\{S_{1},S_{5},S_{6}\right\}$.

## 4. Discussion

### 4.1. Significance Analysis of Influencing Factors on Adoption of Conservation Tillage Technology by Farmers

#### 4.1.1. Personal and Family Characteristics

The age and education level of farmers has a significant positive impact on the adoption of conservation tillage technology, which is consistent with the expected direction. With the increasing of age, the willingness to protect also increases. The reason may be that the older the farmers are, the more experienced they are, the deeper their feelings for the land and the stronger their awareness of protecting the black land. The higher the education level of farmers, the higher the acceptance of new technologies, the easier it is to learn and adopt new technologies and the more able it is to resist unknown risks [58]. Therefore, the higher the education level, the more likely it is to adopt conservation tillage technology.

The amount of the labor force population has a significant negative impact on the adoption of conservation tillage technology, indicating that the smaller the family labor force population, the more the behavior of protecting black soils will be. In the survey, it is found that with a smaller household labor force, farmers will tend to use agricultural machinery operation and technologies such as less tillage and no tillage to reduce the amount of labor.

The gender, whether they have a part-time job and planting years of farmers have no significant impact on the adoption of conservation tillage technology, which is different from the expectation. In the survey, it is not difficult to find that there is not much difference between women and men. They also participate in agricultural work, so it has nothing to do with gender. The black soil region of Jilin Province is relatively cold, and it is basically planted only once a year. Farmers generally choose to go out to work during the slack season in the winter, and most of them are short-term jobs, which do not conflict with the main industry, so it has nothing to do with whether they are part-time or not. The planting years can only reflect the degree of farmers’ mastery of existing technologies, which has no direct impact on the adoption of new technologies, but depends on the cognitive level.

#### 4.1.2. Cultivated Land Characteristics

Planting area and whether cultivated land is scattered have a significant negative impact on farmers’ adoption of conservation tillage technology. The impact of planting area is inconsistent with expectations. The results show that the larger the sowing area, the weaker the farmers’ willingness to protect. Through in-depth communication with farmers, it was discovered that most of the farmers with a large planting area obtained their land through land transfer. They have no long-term contract right to use the transferred land, and the transfer period is different, so it has a significant negative impact on the enthusiasm of protecting black soils. Fragmented and scattered land will seriously restrict the use of agricultural machinery, and require farmers to rush between different cultivated lands, and farmers’ willingness to adopt conservation tillage technology will also be weakened. Therefore, land dispersion has a significant negative impact on farmers’ adoption of conservation tillage technology.

Whether the cultivated land has slopes and the types of crops planted have no significant negative impact on the adoption of conservation tillage technology by farmers. Due to the implementation of China’s policy of returning farmland to forest and grassland, there are few slope lands that are not suitable for farming, and a small slope has little impact on cultivated land. The main crops in Jilin Province are corn and rice. Conservation tillage technology is mainly applicable to dry fields, so it has nothing to do with the crops planted.

#### 4.1.3. Farming Cognition

Whether farmers have received the technical service of conservation tillage has a significant positive impact on farmers’ adoption of conservation tillage technology. This is consistent with the expected judgment. Conservation tillage technology can only be mastered by farmers through the guidance and training of agricultural technology extension personnel. With conservation tillage technology services, farmers will have the ability and peace of mind to adopt conservation tillage technology to protect black soils.

Fertilization measures and whether the following conservation tillage measures are known have no significant impact on farmers’ adoption of conservation tillage technology, which is inconsistent with expectations. Farmers are skeptical about fertilizing measures other than chemical fertilizer. Almost all of them use chemical fertilizer to avoid the risk of yield reduction. They are also skeptical about the specific measures of conservation tillage, so their cognition of conservation tillage technology is not deep enough, and they will be more conservative due to risk considerations, so these two factors are not significant.

#### 4.1.4. External Factors

Whether the government publicity about conservation tillage is known and whether the government’s subsidies for conservation tillage are known have a significant positive impact on farmers’ adoption of conservation tillage technology, which is consistent with expectations. The government publicity will strengthen farmers’ awareness of black soils resources, better recognize the significance of protecting black soils, and increase the enthusiasm of adopting conservation tillage to protect black soils [59]. Conservation tillage subsidies can enhance farmers’ enthusiasm for protecting black soils. The government’s issuance of conservation tillage subsidies to farmers are the most direct way to promote the protection of black soils. Farmers can obtain benefits and will certainly have great enthusiasm for the application of conservation tillage [60].

Whether farmers are satisfied with the local agricultural machinery service has no significant impact on farmers’ adoption of conservation tillage technology, which is inconsistent with expectations. From the survey results, farmers are generally satisfied with agricultural machinery services, so this item is not significant.

### 4.2. Analysis on the Mechanism of Factors Affecting the Adoption of Conservation Tillage Technology by Farmers

**Figure 1:**
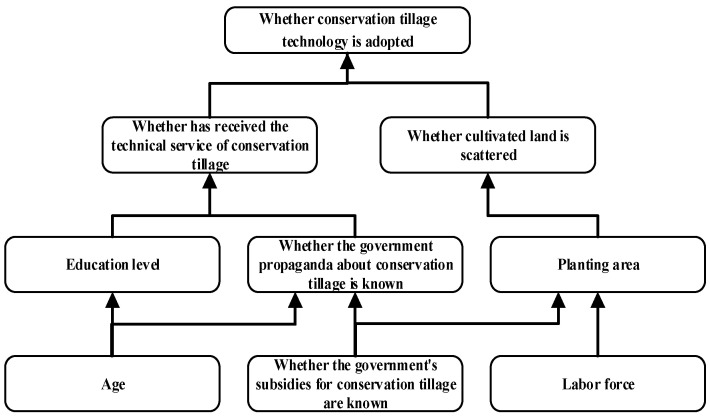
Interpretative structural model of the influencing factors.

The results of interpretation structure analysis show that the eight significant influencing factors of farmers’ adoption of conservation tillage technology are divided into three levels: the first layer includes *S*_4_, whether they have received the technical service of conservation tillage, and *S*_8_, whether cultivated land is scattered; the second layer includes *S*_2_ education level, *S*_3_ whether the government publicity about conservation tillage is known and *S*_7_ planting area; The third layer includes *S*_1_ age, *S*_5_ whether the government’s subsidies for conservation tillage are known and *S*_6_ labor force. Through sorting, the correlation and hierarchy among the influencing factors of farmers’ adoption of conservation tillage technology in the black soil region of Jilin Province, China, are obtained as shown in Figure 1.

It can be seen from Figure 1 that among the influencing factors of farmers’ black soils protection behavior in Jilin Province, whether farmers have received the technical service of conservation tillage and whether the cultivated land is scattered are the direct factors on the surface; education level, whether the government publicity about conservation tillage is known, and the planting area are intermediate indirect factors; Age, whether the government’s subsidies for conservation tillage are known and the labor force are the deep-rooted factors.

Whether farmers adopt conservation tillage technology can be summarized into the following three paths.

Path 1: age → education level, whether the government publicity about conservation tillage is known → whether they have received the technical service of conservation tillage → whether conservation tillage technology is adopted. In this path, age is a deep factor. Since its reform and opening up, China’s economy has developed rapidly, more and more attention has been paid to education, and nine-year compulsory education has been implemented. Therefore, the younger farmers are more likely to receive higher education, and the older farmers have a stronger sense of trust in the government and are more likely to accept the contents of government publicity. The higher the level of education and the wider the government’s publicity on conservation tillage, farmers are more likely to take the initiative to learn conservation tillage technology. The government will also pay more attention to the technical services related to conservation tillage. After learning conservation tillage technology, farmers will have a higher possibility of adopting conservation tillage technology.

Path 2: age, whether the government’s subsidies for conservation tillage are known → whether the government publicity about conservation tillage is known → whether they have received the technical service of conservation tillage → whether conservation tillage technology is adopted. In this path, age and whether the government’s subsidies for conservation tillage are known are deep-seated factors. In Jilin Province, the government has subsidies for conservation tillage, but some farmers do not care about government policies, so they do not know the specific subsidies. Subsidies are the simplest and most direct promotion factor. People who know the government’s subsidies for conservation tillage will also pay more attention to the publicity of conservation tillage. As mentioned above, the older the age, the easier it is to accept the content of government publicity. The more extensive the government publicity, the more attention will be paid to the technical services related to conservation tillage, which will eventually affect farmers’ adoption of conservation tillage technology.

Path 3: labor force, whether the government’s subsidies for conservation tillage are known → planting area → whether cultivated land is scattered → whether conservation tillage technology is adopted. In this path, the labor force and whether the government’s subsidies for conservation tillage are known are deep-seated factors, The larger the number of working people, the larger the area that can be cultivated. After knowing the government’s subsidies for conservation tillage, farmers will be more willing to contract land and accept the transferred land of other farmers, so as to expand the planting area. The larger the planting area, the higher the possibility of land dispersion, but the scattered land is not conducive to the use of agricultural machinery. The use of agricultural machinery is an important part of conservation tillage, so it will affect the behavior of farmers’ conservation tillage [61].

## 5. Conclusions

Based on the data obtained by the research group and by establishing the logistic ISM model, this paper studies the influencing factors of the adoption of conservation tillage technology by 442 farmers in cities or villages in the black soil region of Jilin Province. Through theoretical combing and empirical analysis, we can draw the following conclusions.

Age, education level, whether the government publicity about conservation tillage is known, whether they have received the technical service of conservation tillage, whether the government’s subsidies for conservation tillage are known all have a significant positive impact. Additionally, the labor force, planting area and whether cultivated land is scattered have a significant negative impact.

Among the eight significant influencing factors of farmers’ adoption of conservation tillage technology, age, whether the government’s subsidies for conservation tillage are known and the labor force are the deep-rooted factors. They affect the following factors, including education level, whether the government publicity about conservation tillage is known and planting area. Whether they have received the technical service of conservation tillage and whether the cultivated land is scattered are the direct factors on the surface.

In the context of ensuring China’s food security policy, the government pays more and more attention to the protection of black soils in Jilin Province, a large grain producing province. As the most basic unit of production and management in the black soil region of Jilin Province, the adoption of conservation tillage technology by farmers directly affects the production efficiency of cultivated land in Jilin Province, which is of great significance to ensure China’s food security. Based on the above analysis conclusions, the following policy implications can be obtained:

First, improve the extension system of conservation tillage technology. The average education levels of farmers are junior high school, and the age of farmers is about 44, which leads to their low acceptance and understanding of new things. Therefore, when the government publicizes and promotes conservation tillage technology and improves farmers’ skills, it should fully take into account the characteristics of farmers. The government should make use of the slack time to actively organize farmers to participate in the training of conservation tillage technology, and establish demonstration sites for on-site guidance by technology extension personnel, thus reducing constraints due to age and education.

Second, improve and implement relevant land policies. On the one hand, the subsidy policy for black soils protection is important. It can be seen from the previous conclusion that whether farmers know that the government’s subsidy for conservation tillage is a deep-seated factor. The grass-roots government should give economic compensation to farmers who use conservation tillage technology to protect black soils and ecology, and should also publicize in place and explain more relevant policies, so that farmers can truly understand relevant policies and improve farmers’ understanding and satisfaction with subsidy policies, and it can encourage more farmers to protect black soils. On the other hand, we should improve the land circulation policy, facilitate farmers to expand the planting area for large-scale management, reduce the dispersion of land contracted by farmers, and then promote mechanized production.

Third, strengthen the ability of rural socialized services. The labor force is one of the deep-seated factors. With the development of urbanization, farmers will be more likely to choose to go out to work. The reduction in labor force makes farmers more dependent on agricultural machinery. Therefore, it is necessary to speed up the construction of rural socialized services, which can provide farmers in the black soil region with seed industry services, agricultural machinery services, financial and insurance services that meet the requirements of conservation tillage, and reduce the cost of farmers’ protection of black soils.

Fourth, strengthen publicity of black soils protection. Farmers’ insufficient awareness of conservation tillage is one of the important reasons for their weak awareness of black soils protection, so they should increase publicity through TV stations, websites, mobile phones, books and other channels, vfigorously publicize the laws and regulations on conservation tillage, widely publicize the subsidy policy of conservation tillage, and fully mobilize the enthusiasm and initiative of farmers to adopt conservation tillage.

## Figures and Tables

**Table 1:** Variable description and descriptive dtatistics.

Variables	Variable Name	Variable Definition	Mean	S.D.	Expected Direction
Dependent variable	Whether conservation tillage technology is adopted (Y)	Yes = 1, No = 0	0.78	0.42	
Personal and family characteristics	Age	Actual age/age	43.91	9.86	+
	Gender	Male = 1; Female = 2	1.41	0.49	+
	Education level	illiteracy = 1, primary school = 2, junior high school = 3, High school or technical secondary school = 4, junior college = 5, Bachelor degree or above = 6	3.17	0.94	+
	Whether they have a part-time job or not	Yes = 1, No = 2	1.54	0.50	+
	Labor force	Actual household labor force population/person	2.51	1.03	?
	Years of planting	Years engaged in farming/year	18.85	9.65	−
Cultivated land characteristics	Planting area	Actual planting area/m^2^	12.60	31.07	+
	Whether the cultivated land has slope	Yes = 1, No = 2	1.52	0.50	−
	Whether cultivated land is scattered	Yes = 1, No = 2	1.36	0.48	−
	Types of crops planted	corn = 1, soybean = 2, rice = 3, wheat = 4, other = 5	1.37	0.61	?
Farming cognition	Fertilizing measures	Chemical fertilizer = 1, organic fertilizer = 2, Straw crushing and returning to the field = 3	1.45	0.70	+
	Whether they have received the technical service of conservation tillage	Yes = 1, No = 2	1.46	0.50	+
	Whether the following conservation tillage measures are known	Less tillage and no tillage = 1, Straw residue mulching = 2, Straw crushing and covering = 3, Deep turning or deep loosening = 4, Ridge farming = 5, Rotate with soybean = 6, No above behaviors = 0	2.44	1.13	+
External factors	Whether the government publicity about conservation tillage is known	Yes = 1, No = 2	1.36	0.48	+
	Whether the government’s subsidies for conservation tillage are known	Yes = 1, No = 2	1.50	0.50	+
	Whether they are satisfied with the local agricultural machinery service	Very satisfied = 1, satisfied = 2, general = 3, dissatisfied = 4, very dissatisfied = 5	2.38	0.93	+

Note: in the expected impact, “+” indicates that it may be a positive impact, “−” indicates that it may be a negative impact, “?” indicates that the impact is unknown.

**Table 2:** Results of the regression model.

Independent Variable	Coef.	Std. Err.	z	*p* > z	Odds Ratio
Age	0.050	0.019	2.57	0.010 ***	1.051
Gender	−0.170	0.303	−0.56	0.575	0.844
Education level	0.370	0.177	2.1	0.036 **	1.448
Whether they have a part-time job	0.508	0.309	1.64	0.101	1.662
Labor force	−0.484	0.156	−3.1	0.002 ***	0.616
Years of planting	−0.027	0.018	−1.48	0.140	0.974
Planting area	−0.029	0.009	−3.34	0.001 ***	0.971
Whether the cultivated land has a slope	0.101	0.300	0.34	0.737	1.106
Whether the cultivated land is scattered	−0.879	0.304	−2.89	0.004 ***	0.415
Types of crops planted	0.441	0.304	1.45	0.147	1.554
Fertilizing measures	−0.008	0.204	−0.04	0.968	0.992
Whether they have received the technical service of conservation tillage	−0.122	0.123	−0.99	0.321	0.885
Whether the following conservation tillage measures are known	0.696	0.306	2.27	0.023 **	2.006
Whether the government publicity about conservation tillage is known	0.735	0.334	2.2	0.028 **	2.086
Whether the government’s subsidies for conservation tillage are known	0.851	0.303	2.81	0.005 ***	2.342
Whether they are satisfied with the local agricultural machinery service	0.182	0.173	1.05	0.295	1.199
constant	−3.088	1.690	−1.83	0.068	0.046
—2 Log Likelihood	317.991				
Pseudo *R*^2^	0.324				
Prob > chi^2^	0.000				

Note: ** and *** are significant at the levels of 0.05 and 0.01, respectively.

**Table 3:** Logical relationship of influencing factors.

A	A	A	A	A	A	A	A	S0
0	0	0	0	V	V	V	$S_{1}$	
0	0	0	0	V	0	$S_{2}$		
0	0	0	A	V	$S_{3}$			
0	0	0	A	$S_{4}$				
0	V	0	$S_{5}$					
0	V	$S_{6}$						
V	$S_{7}$							
$S_{8}$								

## Data Availability

Data sharing not applicable.
